# Supplementary material for: Are carbon emissions trading and green financial instruments synergistic? -Comprehensive quantitative research based on content analysis
Source: PLoS One. 2024 Mar 7;19(3):e0298601. doi: 10.1371/journal.pone.0298601 (PMC10919601; doi:10.1371/journal.pone.0298601)
Supplement: S1 Data — (ZIP) [file pone.0298601.s001.zip › Data/地级市Z_F工作报告.zip!/╔╜╬≈/╜·╓╨╩╨/└┤╫╘╣1⁄2╓┌║┼í╛┬φ┐╦╩2╛▌═°í┐_2015.docx]

各位代表:

现在,我代表市人民政府向大会报告工作,请予审议,并请市政协委员和其他列席人员提出意见。

一、2014年工作回顾

2014年,我市经济发展经历了严峻考验。煤炭市场需求疲软,重化工业效益下滑,投资乏力增速放缓,首季GDP增幅断崖式下跌,经济下行压力持续加大。全市上下坚持稳中求进总基调,以改革激活力、增动力,积极实施煤炭新政、建立权力清单、下放行政实权、加快金融创新、培育新型业态、优化投资环境、提升政府效能等一系列务实举措,统筹稳增长、促改革、调结构、惠民生,全市经济发展逆势而上,社会事业全面进步。

——经济运行稳中有进。全市地区生产总值实现1041.3亿元,同比增长6.8%;规模以上工业增加值420.7亿元,增长7.2%;固定资产投资首超千亿,达到1106亿元,增长16.9%;社会消费品零售总额484.3亿元,增长12.8%;公共财政收入117.5亿元,增长2.1%。主要指标增幅全部位列全省前六,其中五项全省前两位。

——发展质量稳步提升。经济结构发生积极变化,第一产业优势逐步释放,服务业占比提升2.3个百分点、达到42.6%,成为拉动经济新引擎;新兴工业投资占到62.6%,非煤产业增加值比重提高2.5个百分点。单位GDP能耗下降3.89%,减排任务全面完成,新增造林50万亩,空气质量明显改善。108廊带获批全省唯一区域一体化发展示范区,县域经济发展提质加速。

——人民生活持续改善。全市居民人均可支配收入17213元,增长9.5%,GDP含金量全省前列,其中农民收入增长11%,增速连续四年超过城镇居民,半数县份过万元。全市城镇新增就业3.83万人,转移农村劳动力3.9万人,登记失业率1.94%。CPI涨幅1.6%,低于控制线1.9个百分点。教育、卫生、文化、社会保障等基本公共服务水平取得新提升。

我们攻坚克难,主动作为,不辱使命。

(一)精准发力,保持经济稳定运行。强化经济运行分析调度,牢牢抓住“煤炭、企业和项目”三个关键,大胆出手,定向施策,着力解决机制创新“抢先一公里”和政策落实“最后一公里”的问题。率先出台清费减负、收储包销、代料洗选等“煤炭十条”,促进煤矿企业稳产增效,稳定了经济基本面。全年生产原煤8979万吨,增长6.6%,产销率全省第一。优化企业发展环境,市县两级同步晒出权力清单,市本级5175项涉企行政权力“挂起”三分之二,建立首违不罚、容期整改等十项制度,把权力关进制度的笼子。强力推进项目建设,创新建立项目联审代办等服务机制,引深“百日百项达标行动”,推动766项重点项目完成投资过千亿,“六位一体”进入全省中上游水平。全市经济止缓回稳、逐季上升、稳步向好。

(二)培优扶强,推动产业转型升级。以煤电一体化、农业现代化、旅游集团化、业态新型化为主攻方向,放大优势,挖潜增效。五个低热值煤发电项目获得路条,居全省首位,晋电送冀项目成功落地,“十三五”可新增装机1500万千瓦,极大优化我市工业结构。现代农业发展加速,我市入选国家现代农业示范区,金谷农投规模运营、技术服务、股权投资初见成效,全市农业龙头企业营收180亿元,淘宝晋中馆上线交易,果树政策性保险率先试点,农技推广体系逐步完善。新兴服务业势头强劲,与央企国旅签署合作协议,将通过聚合资本、整合资源,促进旅游产业做大做强;乔家大院5A景区挂牌,石膏山、红崖峡谷成功创建4A景区,平遥古城生态旅游文化产业园项目落地,云竹湖景区建设加快,全市旅游总收入突破400亿元,跨入中国旅游城市六十强;太铁物流中心开建,54项文化产业项目完成投资18亿元。提升传统产业,焦化行业完成兼并重组,工业新型化“百强项目”完成投资145.4亿元,吉利新能源汽车生产线四大工艺设备安装基本到位,太钢万邦高碳铬铁等37项标志性项目投产,市级产学研创新服务平台初步建成,园区承载能力进一步增强。

(三)创新引领,破解改革发展难题。把改革作为稳增长、促发展的关键举措,啃了不少硬骨头,破了一批老难题,持之以恒打基础利长远。金融创新全省领先,天交所山西运营中心落户,我市挂牌企业占到全省三分之二,五户企业在上海股交中心集体挂牌,居全省第一;在全国首创产业“升级贷”,平遥、介休农信社成功改制,瑞阳供热首开全省资产证券化融资先河,平遥日升隆首发全省第一单私募债,全市新增贷款增幅全省第一。土地利用日趋高效,用足用活土地政策,落实土地周转指标3万亩;盘活优化存量土地5465亩,占全市供地总量39.6%。强化预算硬约束,全面推行预决算公开,创新实施项目投资评审,1971个项目审减资金98.7亿元。大力推广PPP模式,323亿元社会资本进入30项城市公建项目。建立“土地银行”和农村产权交易中心,全市土地流转率27%。市城区城市管理体制实行建管分离、综合执法,介休市城市管理行政权集中试点取得成效。太谷、祁县农村改革全国领先。国有企业、户籍制度、食药监管、工商便利化等改革进一步深化。

(四)扩容提质,统筹推进城乡建设。大力实施“一区两翼”发展战略,城镇化率提高1.58个百分点,达到50.45%。城市面貌进一步改观,城区坚持“新区连片大开发、旧区提质大突破”的思路,攻克了一批群众期盼亟需解决的难事,4大片区、5个棚户区、14个城中村启动改造,征拆面积31万平方米,一批安置小区主体接近完工,大学城商业配套初具规模;锦纶路快速化改造和乌金山旅游专线等25条道路建成,“三馆一院”建设进展顺利、今年底封顶,规划馆开馆迎客,百草坡公园主体完工;人行天桥实现“零”的突破,道路微循环、路口渠化、公交港湾使出行更加通畅,市城区重点工程完成投资127.2亿元。城乡基础设施日益改善,大西客专六个站前广场和通站路建成投用,我市进入高铁时代;阳黎高速阳泉至左权段通车,实现县县通高速;松塔、恋思新水源工程开始供水,东山供水、中部引黄两大水网工程顺利推进,群众饮水安全更有保障。人居环境改善提升,大县城、示范镇建设完成投资167亿元,介休被确定为全省唯一的国家新型城镇化综合试点、并进入国家卫生城市行列,市城区连续7年蝉联省级卫生城市,平遥、祁县荣列省级园林城市,太谷成为省级林业生态县,“四大工程”让农村更加整洁靓丽。在征地拆迁、道路改造、城市建设过程中,人民群众给予了极大的包容、理解和支持,更加坚定了我们建设好、管理好这座城市的信心和决心。

(五)优化服务,努力改善民生民利。全市民生支出占公共财政预算支出83.8%。就业政策更加积极,出台三个“保就业”意见,政府购买基层服务、公益性用工优先向困难群体和服务岗位倾斜,成功举办首届“晋商杯”大学生创业大赛。社会保障能力增强,城乡居民养老保险并轨,各类社保补助提标全部到位,建成农村老年人日间照料中心195个、其中昔阳建成90个。社会事业健康发展,7个县区通过国家义务教育均衡县认定,市城区学校三年建设规划启动、6所开建,全市新建改造幼儿园52所;县级公立医院改革扎实推进,市内和省城异地就医实现即时结算,群众看病更加方便;全民文化行动全面实施,500米健身圈全覆盖;农村“五件实事”完成年度任务,又有3.2万贫困人口脱贫;保障性住房建成1.76万套,农村危房改造1万套,在全省率先实施并完成400人以上自然村通达水泥(油)路工程。社会治理不断创新,安全生产经营性事故起数和死亡人数“双下降”。全面推进精神文明建设,平安稳定和谐局面进一步巩固。档案、科普、人防、气象、史志、外事、侨务、对台、援疆和民族宗教等工作不断加强,妇女儿童、老龄、红十字会、残疾人事业健康发展。

(六)革弊立新,提升政府服务效能。深入开展党的群众路线教育实践活动和“学习讨论落实”活动,加快服务型效能政府建设。推进“六权治本”,建立财政供养人员用人管理机制,社会资源公开拍卖出让,强化国有企业绩效管理,推行商业地产开发项目招商招标一体化竞争,完成“两集中、两到位”改革,这些措施很大程度上消除了权力设租寻租空间,减少了权力滥用。推进民主法治建设,自觉接受人大法律监督和政协民主监督,以解决问题为标准,办结人大代表建议253件、政协提案296件;启动法治晋中三年行动计划,政府法律顾问制度全覆盖。优化行政流程,对政府职能转变、行政审批体系、公共财政支出管理进行顶层设计,重新释权分配,率先完成政府系统机构改革;以基层需求为导向下放实权,取消下放行政审批项目108项,向开发区下放权力13项、委托权力1153项,政府采购、重大投资、项目预算引入第三方评估。坚决抵制“四风”,政府系统工作实行年中和年终两次绩效评价,全面加强行政监察、审计监督和政务督查,建立工作差错通报制度,政风行风明显改观。

过去一年,我们探索实施了一批破题性改革,发展环境优化,行政效能提升;强力推进了一批重大项目,经济基础稳定,发展后劲更足;用心办成了一批惠民利民实事,人民更加幸福,社会更加和谐。这是省委、省政府和市委正确领导的结果,是全市人民团结奋斗的结果。在此,我代表市人民政府,向全市广大干部群众,各民主党派、工商联和人民团体,向驻地部队广大指战员、武警官兵、公安民警和国家、省驻地单位,向所有关心、支持、参与晋中改革发展的各界人士,表示崇高的敬意和衷心的感谢!

同时,我们必须清醒地看到发展中存在的问题:一是部分指标未完成考核目标,反映出我市经济结构单一,产业链条较短,企业规模偏小,抗风险和盈利能力较弱,政府应对复杂形势的能力亟待提高。二是城市建设、公共服务、社会保障、安全生产、食药安全等领域还存在薄弱环节,征地拆迁、环境污染等问题群众反映较大。三是政府职能转变、政务环境优化还不到位,公务人员观念、作风、素质与社会要求和人民期盼还不适应。四是明哲保身、庸懒怠政、“四风”问题和一些领域腐败现象仍然存在。对此,我们一定要下大力气认真解决,绝不辜负全市人民的期望!

二、2015年工作安排

2015年,是全面深化改革的关键之年,是全面推进依法治国的启动之年,是全面完成“十二五”规划的收官之年,也是我市加快推进“六大发展”的开局之年。

当前,世界经济复苏缓慢,我国经济进入“增速换挡、结构调整、动力转换”新常态,经济下行压力持续加大。特别是我市受煤炭需求下降和价格持续下跌的双重挤压,煤炭企业困难加剧;受产业升级缓慢和产品优势不足的双重约束,原材料和加工制造企业订单减少、开工不足、效益下滑,困难远比去年更大。我们必须抓住新机遇,在“一带一路”、京津冀一体化、晋中城市群、山西科创城等战略机遇中找准定位、主动作为;必须用好新优势,大力实施“六权治本”,加快推进“六大发展”,净化政治生态,创优发展环境,促进富民强市;必须适应新常态,全面推进以“权力瘦身、效能提升、企业减负、市场主导”为主要内容的效能革命,以项目提质增效加速为工作主线,坚定信心,迎难而上,实现更好更快发展!

做好今年政府工作,要以党的十八大、十八届三中、四中和省委十届六次全会精神为指导,以“四个全面”战略布局为统领,深入学习贯彻习近平总书记系列重要讲话精神,主动适应经济发展新常态,坚持稳中求进工作总基调,以提高经济发展质量和效益为中心,转方式调结构促改革,严格落实党风廉政建设主体责任,全力推进“六权治本”和“六大发展”,着力保障改善民生,促进全市经济社会平稳健康发展。

2015年经济社会发展的主要预期指标是:地区生产总值增长7%,规模以上工业增加值增长7%以上,固定资产投资增长16%以上,公共财政收入增长2%以上(剔除过渡户清理和财政两权收入减收等因素,同口径增长15%以上),社会消费品零售总额增长12%,城镇、农村常住居民人均可支配收入分别增长8%以上、10%以上;新增就业岗位3.6万个,城镇登记失业率控制在4.2%以内,居民消费价格涨幅控制在3%以内。

约束性指标是:万元地区生产总值综合能耗和二氧化碳排放量分别下降3%左右、3.7%,污染减排六项指标完成省定任务,万元工业增加值用水量下降3.2%。

实现上述目标,既要全面统筹又要重点突破,要加快推进108示范区、清洁能源基地、科技创新高地建设,重点突出项目提质增效加速、国家级现代农业示范区建设和文化旅游现代服务业攻坚,扎实做好八方面工作:

(一)加快转型综改区建设,全力推进改革创新。引深“转型综改攻坚年”活动,实施“233”年度行动计划,选准“五个突破口”破解瓶颈制约,持续释放改革红利。

以基础设施提升为突破口,有效推进108示范区建设。落实“三年初见成效”要求,按照“基础提升、重点强化、反向扶持、培育特色”思路,分阶段按步骤扎实推进。完成五年建设方案和三年行动计划,细化任务分解责任。重点抓好108国道沿线综合改造、美丽乡村建设、生态修复、汾河整治等标志性工程。做好产业布局、土地管理、财税体制、民生保障等顶层设计,促进资源要素一体化配置,推动区域协调发展。

以PPP模式推广为突破口,创新投融资机制。大力推广PPP模式,优化多元投资环境。建立市本级鼓励社会投资项目库,引导更多社会资本进入公共领域,加快推进城市轨道交通、汇通大厦和晋中综合环境循环经济产业等PPP合作项目。加快金融改革创新,巩固完善政银企合作长效机制,切实增强对实体经济的融资支持,培育优势企业;依托天交所山西运营中心培育优良上市资源,推动上海股交中心山西基地落户我市,实现在上海股交中心、“新三板”挂牌和联交所上市三个“零”的突破;发展融资性担保机构,建立企业资金链应急周转保障资金,设立昔阳、和顺村镇银行,完成左权农信社改制,加快发展资产证券化业务,积极有效防范金融风险。

以预算科学公开为突破口,深化财政体制改革。政府收支全部纳入预算管理,启动中期财政规划管理,开展三年滚动预算编制试点。严格预算执行,实施税务策划,试点开展部门整体支出绩效评价,优化政府采购,严控政府债务,让公共财政资金用得科学、花得透明、看得明白,真正把有限的资金用在产业发展、基础提升、民生改善的刀刃上!

以强化绩效管理为突破口,提升国有资产收益。加大国有企业和经营性事业单位改革力度。完善国有资本监管体系,实行国有企业任期绩效考核,严格对标评价,结果与企业负责人薪酬和选聘任用挂钩,确保市本级国有资产经营收益超过同行业平均水平。财政资金要重点投向民生公共服务、战略性新型产业、生态环境、科技进步等领域,退出其它竞争性开放领域,切实提高公共资金使用效益。

以盘活存量土地为突破口,创新土地管理机制。用足用好土地三项改革政策,创新土地出让定价机制,加强土地供后监管,依法清理收回批而未供和长期闲置土地,采取回购出让、作价入股等方式二次高效利用,做实土地收储供应,确保全年2.6万亩项目用地供给。加快土地利用总体规划调整完善,年底基本完成农村宅基地和集体建设用地地籍调查工作。加快推进不动产统一登记制度改革。

我们要拿出“逢山开路、遇河搭桥”的勇气,破解发展难题,增强发展活力,加快走出资源型地区可持续发展的新路子!

(二)推动项目提质增效加速,全力促进产业升级。深入开展项目“提质增效加速年”活动,以项目带动投资,以投资促进转型,以转型加快发展。

做实项目盘子。坚持激活存量、优化增量,深入挖掘项目线索,主动对接项目主体,全面梳理2012年以来签约未落地的存量项目,创造条件加快推进。完善招商引资分类目录和招商条件,强化招商引资平台作用,实行快速服务流程,加快要素供给,推动项目落地,持续保持投资后劲。

做优项目结构。坚持扶优汰劣、腾笼换鸟,重点推进五大类十大重点领域1060亿元投资,力争全市新兴产业项目投资占比提高8个百分点,服务业增加值比重提高1个百分点,煤炭就地转化率提高5个百分点。推进煤电化基地建设,全面开工5个低热值煤发电项目,完成河北建投寿阳2×100万千瓦发电项目前期,抓好已核准的6个风电、光伏发电项目;煤化工产业重点抓好平遥煤化天然气、阳煤寿阳乙二醇和昔阳煤电化工业园区项目建设。落实“中国制造2025”战略,大力发展高端装备制造业,加快建设山西新能源汽车城,吉利新能源汽车力争实现量产,北达发动机项目全面开工,争取电动客车项目落地;改造提升冶金、液压、玛钢、纺机产业,支持祁县培育壮大食品、玻璃器皿等消费品产业,建设山西消费品产业隆起带和集聚区。积极推进东方希望铝系综合循环经济园项目,力争年底100万吨氢氧化铝生产线建成投产。大力发展新型材料,做大医药产业,依托创新城、大学城年内启动建设开发区科技园。

做好项目服务。坚持结果导向、跟踪问效,实行重点工程现场月调度制度,落实审批代办、团队服务机制,加强重点监测项目常态化、精细化管理,促进“六位一体”落到实处。严格政府投资项目行为审批和方案审批,完善代建制和项目法人制,加强中介机构管理,提升项目前期和工程建设效率。突出投资主体和政府部门两个责任,建立项目建设评价追责机制,严格奖惩兑现,确保部门服务到位、项目投资主体履约到位,全面完成年度投资任务。

今天的项目就是明天的速度、结构。我们要全力推进项目提质增效加速,增强拉动能力,促进产业转型!

(三)推进煤炭革命和企业振兴,全力稳定经济基本面。牢牢抓住支柱产业、优势企业和重点领域,突出“三个创新”,激发内生动力,确保经济运行平稳健康。

创新机制,推动煤炭产业升级。加快煤炭“六型”转变,推进煤炭管理体制、监管方式改革,切实减轻企业负担,引导鼓励企业签订长期供需协议,提升煤炭附加值,扩大就地转化能力。完善产销信息共享平台和税收稽查监管机制,加快形成煤炭营销新秩序。加大煤层气开发力度,实现高瓦斯矿井瓦斯抽放全覆盖,抽采利用率达到40%以上。加快安全高效矿井建设,新建现代化矿井3座,所有生产矿井安全生产质量和管理全部达标。

创新服务,帮扶企业健康发展。加强经济运行监测、预警、调度,加快市县两级中小企业窗口服务平台建设。继续松绑激活,严查违规行政损坏企业利益的行为,不折不扣落实省政府企业减负60条措施,减轻企业负担40亿元以上。加大政府采购本地产品力度,定向开辟甲控材市场。大力推进中小微企业和民营经济成长工程,全面完成民营经济改革试点任务,加快工商登记制度便利化改革,优化创业环境,激发市场主体活力。

创新供给,引导消费快速增长。提升高校新区商业服务配套能力,加快建立多层次、多领域、广覆盖的生活服务体系,形成规模消费。发挥科创城、大学城、基础教育、职业教育、文化体育等品牌优势,推动相关领域的合作与渗透,吸引就业创业和人口集聚,创新引导消费需求,加快培育新型消费业态。培育发展“互联网+”经济新模式,优化和集成要素配置。完善农村市场体系,农家店商品统一配送率达到50%,完成600家农家店信息化改造,农村市场消费增长16%。

(四)实施文化旅游开发攻坚,全力推动服务业提档升级。落实促进服务业发展的政策措施,加快生产生活性服务业发展,重点培育文化旅游和现代物流产业。

做强文化旅游产业。开展“旅游攻坚年”活动,重点实施管理体制改革攻坚,落实与国旅集团的合作开发项目,完善旅游业管理、投资、服务和营销平台功能;实施基础设施建设攻坚,加快10条旅游公路改造,新建一批高标准停车场、旅游厕所;实施品牌宣传营销攻坚,全面开发东山生态休闲旅游,做大做强平川晋商文化旅游,扩展大西高铁冠名“晋中号”效应,启动京广、京沪高铁宣传,扩大晋中旅游品牌影响力;实施环境秩序整治攻坚,突出4A以上重点景区,净化旅游生态;实施景区提质升级攻坚,围绕游客个性化需求,强化链条打造,整合资源线路,创新产品供给,重点推进平遥古城生态文化产业园、祁县千朝农谷、榆社云竹湖、左权龙泉、百草坡房车营地等45个景区提质项目,平遥古城挂牌5A景区,王家大院加快5A创建,大寨景区启动5A创建,再创一批3A级以上景区;实施文化旅游融合发展攻坚,推进山西传媒文化创意产业基地等64个文化产业重点项目,促进文化旅游资源优势向竞争优势、发展优势转变,全年旅游总收入完成460亿元,为“十三五”末建成千亿产业奠定基础。

做大现代物流业。发挥交通、区位优势,重点推进太铁货运物流中心项目,年内完成60%以上的工程量,打造中西部地区公铁联运、综合性、现代化物流中心。推动苏宁物流配送、中储、经纬等物流项目建设,建立和完善现代物流服务体系,着力提升物流业对相关产业的综合服务能力。

(五)提升现代农业水平,全力做好“三农”工作。挖掘释放晋中农业产业发展的传统优势,围绕“四条路径”持续发力,促进农村发展、农业增效、农民增收。

在做大特色优势产业规模上发力。全面启动国家现代农业示范区建设,统筹规划,加快推进“两县六村一企十园”建设。做优“一村一品”特色,稳定粮食生产,新发展设施蔬菜6万亩、干鲜果13万亩,新改扩建标准化养殖园区100个,畜禽饲养总量突破1亿头(只)。创建太谷、祁县两个省级农产品质量安全示范县,全市新规范认证“三品一标”面积1万亩,农产品“三品”认证12个。强化农业科技普及推广,完成330个村级农技服务点建设,解决好农技推广最后一公里问题。主要粮食作物综合机械化水平达到74.4%。

在创新经营方式上发力。推进农业集团化经营,发挥金谷农投集团的引领作用,大力发展资本农业、科技农业、品牌农业,设立运营现代农业产业基金和担保公司,放大资金杠杆作用,破解农业融资难题;太谷现代农业科技创新园建成运营,组建科技服务公司,输出先进技术、科技品种、产品检测等服务。创新农产品流通方式和业态,完善城乡流通基础设施和市场体系,完成农产品现代流通示范园区建设,启动国家肉类蔬菜流通追溯体系试点城市建设,做好“线上线下”流通结合,提升“淘宝晋中馆”运营水平。壮大农产品加工龙头企业,销售收入增长12%以上。

在全面深化农村改革上发力。深化农村土地制度改革,全面开展农村土地承包经营权确权登记颁证工作,加快县级产权交易中心建设,用活用好土地流转专项资金,有序推进农村土地流转,积极扶持规模经营。探索推进农村集体产权制度改革,扎实开展农村土地征收、集体经营性建设用地入市、宅基地制度改革试点。创新农业经营主体,新培育30个省级、50个市级、80个县级专业合作示范社和30户市级家庭农场,规范扶持发展60个生态庄园。统筹实施农村综合改革,祁县启动国家级农村改革试验区试点工作,太谷完成国家现代农业示范区农业改革与建设试点任务。大力推进水利、国有林场改革。积极稳妥、有序推进供销社综合改革。扩大政策性保险覆盖范围,全面推开果树保险试点工作。

在产业扶贫开发上发力。坚持开发式扶贫方针,以673个贫困村为主战场,以精准扶贫为抓手,加大产业扶贫、技能提升、金融扶贫力度,产业扶贫“双百”工程完成投资20亿元,就业培训3800人,确保20个贫困村和3.97万贫困人口脱贫。

(六)加速晋中城市群建设,全力提升新型城镇化水平。以晋中城市群建设为引领,建管并举,城乡统筹,实现新型城镇化建设的“三大提升”,全市城镇化率再提高1.6个百分点左右。

面子里子齐抓,提升城市功能。创新投融资模式,撬动社会资本,加快城市建设速度。大力改善城市人居环境,重点推进“公共服务、出行畅通、生态文明、设施提质、住房改善”五大惠民工程,完成投资120亿元。着力推进北部新城、片区改造等一批连片整体开发工程,以太平洋绿色森活城、“三馆”、大学生文化娱乐中心为标志,全面铺开北部新城开发;加快5个片区、16个棚户区及20个城中村改造,力争三年内完成城中村改造。着力推进路网改造、管网建设等一批功能提升工程,完成龙湖大街—锦纶路—思凤街—汇通路内环道路快速化改造,力争迎宾街至科创城、大学城至太原南站两条城市轨道交通项目开工建设,新建20座人行过街天桥,形成基本满足交通便捷化需求的城市快速路网体系;加大市政建设力度,供水、供热、供气普及率和生活污水处理率完成“十二五”任务。着力推进公园绿地、公共交通、社会保障等一批民生重点工程,晋中大医院主体完工,晋商公园三期等5个公园建成开放,加快潇河水上游乐园等3个主题乐园建设,高标准推进市殡仪馆和公墓建设;加快公交扩容和公共自行车服务系统建设,完成10条主干道60座公交站智能化改造,增加公交车辆,社会化投资公共自行车站点250个、车辆5000套。

建设管理并重,提升城市形象。创新城市管理理念,借智借力合作伙伴,提升城市建管水平。强化规划编制管理,完善城市基础功能专项规划,市城区控规实现全覆盖,介休市达到50%。公开商业地块开发规划条件,充分市场竞争,加快城市建设。深化环境综合整治,昔阳加快国家卫生县城创建,左权、灵石通过国家卫生县城复查;市城区重点实施中都路、安宁街综合整治,通过省级文明城市验收。全面推行城市管理综合执法,推广介休、昔阳城市管理经验,年内开通数字城管系统,确保城市管理全天候全方位保持受控状态。市城区公开拍卖公共停车资源经营权,实施9项城市交通智能化管理项目,大力解决拥堵问题。

城乡一体推进,提升统筹水平。全面开展城乡爱国卫生清洁运动,推进15个国家级重点示范镇“五建设两整治”工程,全市所有县城、乡镇、村庄全面启动卫生创建工作,高标准建设100个美丽宜居示范村。推进我市国家中小城市综合改革试点和介休国家新型城镇化综合试点建设,加快昔阳巴洲城镇化示范园区等重点小城镇建设。加快推进农村人居环境改善工程,完成投资20亿元以上。优化城乡交通条件,改造提质农村公路500公里、新增县乡公路50公里;加快推进和邢、阳大铁路建设和晋衡高速前期,盂榆线路面大修和省道319左权过境改线工程竣工。增强水支撑能力,协调推进东山供水、中部引黄两大水网建设,力争受水县小水网工程开工。建设平遥1000千伏特高压变电站、太谷贯家堡220千伏、昔阳新城110千伏变电站等工程,继续推进农网升级改造和低电压治理。扎实办好“五件实事”,改造农村困难家庭危房6000户,新建改建农村幼儿园33所,易地扶贫搬迁5000人,深入推进乡村清洁工程,培训新型职业农民1万人。加快户籍制度改革,全面放开城镇落户条件,统一城乡户口登记制度。

(七)加强生态文明建设,全力打造美丽宜居新晋中。要突出“三个强化”铁腕治污。

强化节能降耗攻坚。加强节能预警调控,推行能效对标。加大节能执法监察力度,积极推广合同能源管理和节能技术改造。实施工业能效提升计划,推进余热余压利用等重点节能工程和项目。扶持重大资源综合利用示范项目,推动粉煤灰、煤矸石等工业大宗固废资源化利用,工业固废综合利用率达到65%。

强化污染减排达标。严格执行环境保护法,突出“控煤、治污、管车、除尘”四个关键环节,深入开展电力行业脱硫、脱硝、除尘改造,全力推进燃煤电厂超低排放提速工程,全市所有焦化企业完成脱硫提标升级改造,推进6个县城污水处理设施提标改造。实施燃煤锅炉综合整治攻坚战,力争年内彻底解决市城区燃煤锅炉污染问题,加快淘汰黄标车、老旧车,PM2.5浓度再下降2%。加强重点流域水污染防治,地表水达到省考核要求,饮用水源地水质全部达标。加强农业源污染治理,现有规模化养殖场治理率要达到60%以上。创新环保投入机制,通过PPP、特许经营等模式修复生态、发展环保产业,试行第三方治理。创新监管方式,完善举报奖励制度,构建群众参与和网格化监管相融合的环境监察体系。固化专项整治效果,确保不发生环境污染事件。

强化生态环境建设。以创建省级林业生态市为抓手,重点实施25项市级重点造林绿化工程,完成新造林合格面积30万亩,林木绿化率增加1个以上百分点,左权、昔阳争创国家园林县城,平遥建成省级林业生态县。强化森林资源保护,新建林区防火通道400公里,大力提升森林防火综合能力。开展新一轮退耕还林还草工程。统筹推进水土保持、水生态保护和湿地保护工作,启动境内汾河流域生态修复规划,推进3个国家水土保持重点县建设。完善生态补偿机制,大力推进采空区、沉陷区、煤矸石山的土地复垦和生态修复,实施好5县240户地质灾害避让搬迁治理任务。

环境是最普惠的民生。我们要像保护眼睛一样保护生态环境,让绿水青山蓝天成为晋中发展新优势!

(八)全面发展社会事业,不断改善民生民利。要坚持民生导向,按照兜底线、抓重点、补短板、建机制思路,实施一批顺民心、谋民利、造民福的实事,让人民在改革发展中得到更多实惠,增强人民群众的获得感。

稳定扩大城乡就业。加大对困难企业帮扶力度,“五缓三补”政策期限再延长一年,落实好失业保险支持企业稳定岗位政策。着力实施高校毕业生创业引领计划和就业促进计划,办好第二届“晋商杯”大学生创业大赛,积极推进创业带动就业。全力构建和谐劳动关系,落实农民工工资保证金和政府应急周转金制度,企业劳动合同签订率达到98%,努力做到不欠农民工辛苦钱。

加快教育均衡发展。实施第二轮学前教育三年行动计划,新改扩建17所公办标准化幼儿园,全市基本普及学前三年教育。推进义务教育发展基本均衡县创建工作,昔阳、和顺、祁县、平遥4个县通过国家评估认定,率先在全省实现县县达标。统筹发展高中阶段教育,大力实施高中标准化建设工程,着力构建中职高职沟通、职前职后衔接的现代职业教育体系,培养我们自己的“蓝领”。认真落实特殊教育提升计划,全市残疾儿童义务教育入学率达到85%以上。加快推进市城区6所学校建设,力争再启动4所。扎实抓好全国教育信息化改革试点工作。

多渠道增加居民收入。推进机关事业单位工资制度改革,落实县以下机关公务员职务与职级并行制度。推动企业工资集体协商,严格执行最低工资标准和带薪休假制度。大力引导农村劳动力转移就业,建立健全征地拆迁补偿机制,创新城中村改造生产生活安置机制,依法保障农民合法权益。

提升社会保障水平。实施机关事业单位养老保险和职业年金制度改革,落实企业职工基本养老保险统收统支办法,城乡居民基础养老金标准人均月提高15元;完善城乡居民大病保险制度,实现省内异地就医直接结算,城镇居民医疗保险年最高支付限额提高到40万元,城镇居民基本医保和新农合人均财政补助标准提高到380元,真正让人民群众看得好病、看得起病。全面推进“五险统征”和社保“一卡通”,完成各项社会保险扩面及征缴任务,扎实推进全国养老服务业综合改革试点工作,新改扩建农村和城市社区老年人日间照料中心110个,为农村60岁以上老人免费体检。做好残疾人康复救助工作。全面实施临时救助制度,确保困难群众求助有门、受助及时。推进公租房与廉租房并轨运行,城镇保障性安居工程开工16865套,建成1.28万套,完成投资40亿元。

深化医药卫生体制改革。县级公立医院改革实现全覆盖,人均基本公共卫生服务经费财政补助标准提高到40元。加强村医队伍建设和卫生人才培养,推进资源下沉,实行分级诊疗。加强基层医疗机构基础建设和公共卫生、疾病防控工作。

发展文化体育事业。大力培育和践行社会主义核心价值观,大力弘扬“三个文化”和新时期晋中精神。深入推进“全民文化行动”,继续实施文化惠民工程,做好文物古建和非物质文化遗产保护,启动晋中大剧院、群艺馆、妇女儿童活动中心、老干部活动场所等改造,深化市直文艺院团改革。加大文化艺术创作支持力度,做好扫黄打非和净化网络工作。扎实开展全民健身系列活动,办好第四届市运会,筹建市级游泳馆和综合训练馆,健全城乡体育场地设施管理维护机制,推行体育场馆免费开放,引导发展户外营地等新型体育产业。

深入推进创新驱动。强化互联网思维,打造产学研一体化高地,全面推进科技园建设,加快山西卫星信息应用、山西产业研究院等一批高科技项目入园建设,促进“大学城”向“科学城”转型。加大知识产权保护力度,实行科技人员股权、期权、分红鼓励政策,完善市级科研技术交易等服务平台,完成“5个1”科技支撑任务,推进科技孵化基地、院士和博士后科研工作站、产业技术创新联盟、工程技术中心等创新载体建设,发挥科技支撑产业升级转型的核心作用。

加强和创新社会治理。扎实推进“平安晋中”建设,全力打造立体化防控体系。加强社会治安综合治理,深入开展“打黑除恶专项斗争推进年”活动和社会治安“六项整治”行动,严厉打击各类犯罪活动,防止群体性事件和群死群伤事故发生。加强网格化管理和基层综合服务管理平台建设,落实重大决策社会稳定风险评估机制,建立完善多元化矛盾纠纷调解机制。把信访纳入法治轨道,及时解决群众合理诉求。强化食品药品安全监管,确保人民群众饮食安全、用药安全。加强应急管理和防灾减灾能力建设。

支持国防和军队建设,做好双拥、优抚安置和人防工作。发展妇女儿童、老龄和红十字会等事业。做好民族宗教、外事、侨务、对台、人口计生和援疆等工作。加强气象、地震、科普、档案、史志等工作。高标准做好“十三五”规划编制工作。

千方百计抓好安全生产工作。牢固树立底线思维和红线意识,全面落实党政同责、一岗双责,加大技术兴安力度,危险化学品、烟花爆竹和规模以上冶金等工贸企业标准化建设100%达标,提升本质安全水平,加快创建安全发展先进城市。按照“全覆盖、零容忍、严执法、重实效”要求,扎实开展隐患排查、打非治违等专项行动,全面加强煤矿、道路交通、非煤矿山和尾矿库、水库、人员密集场所以及建筑施工、油气管道、城市燃气、特种设备等各行业各领域的安全生产工作。积极筹建96333电梯安全监管应急处置平台。坚决杜绝重大以上事故,遏制较大事故,减少一般事故。

安全生产是做好一切工作的前提和基础。我们要高度重视、警钟长鸣、常抓不懈,确保实现安全生产形势持续稳定好转!

三、切实加强政府建设

要解放思想,开阔视野,转变职能,提升政府治理能力和服务水平。

坚持依法行政,建设法治政府。全面推进“六权治本”,依法确权、科学配权、制度束权、阳光行权,严格权力监督,强化规矩纪律意识,坚持“法无授权不可为、法定职责必须为”,严格执行权力清单和责任清单制度,把权力关进制度的笼子。自觉接受人大法律监督、政协民主监督和社会各界监督,认真办好代表建议、委员提案,对人民群众负责。严格执行决策制度和决策程序,强化重大决策失误责任追究,确保决策科学民主合法。加强法治宣传教育,做好法律服务和援助工作,完成“六五”普法任务。

提升服务质量,建设效能政府。以敬民之心,行简政之道。加快职能转变再造行政流程,不断提升行政效能和财政绩效。以基层需求和群众需要为导向,再取消下放一批行政审批事项,全面清理非行政许可审批。加快整合市级公共资源交易平台,规范交易行为,减少权力干预。大力整治“红顶中介”,严查中介机构强制服务、过度服务。加大政府系统工作绩效评价,逐步建立末位请辞制度。深入推进四级政务服务体系标准化建设,加快建设综合政务服务平台,让群众办事更加便捷。

大胆解放思想,建设创新政府。改革创新是发展潜能的“挖掘机”。各级政府和公务人员要树立创新意识,敢于打硬仗,善于过险滩。自觉学习新知识,研究新问题,打破旧习惯,建立新模式,切实提升履职能力。始终坚持问题导向,瞄准影响经济社会发展的全局性问题、制约转型升级的突出矛盾和群众反映强烈的民生诉求,有效破解障碍和瓶颈。激发改革创新的热情,真正做到探索无禁区、改革无禁区,以政府的创新作为推动全民创业、万众创新。

从严惩治腐败,建设廉洁政府。不折不扣落实党风廉政建设主体责任和“一岗双责”,健全廉政风险防控机制。加强行政监察、审计监督和政务督查,拓展第三方评估领域,深化政务公开,坚决打掉寻租空间。深入开展“三严三实”专题教育,扎实推进学习讨论落实活动专项整治,切实解决突出问题。落实“约法三章”,“三公”经费只减不增。实行权力运行纠错问责和考核追究制度,大力治理“庸懒散浮拖”。坚持有腐必反、有贪必肃,切实做到为民务实清廉。

各位代表!改革发展富民强市的任务艰巨而光荣。让我们在省委、省政府和市委的坚强领导下,团结全市人民,锐意进取,求真务实,推动我市经济社会更好更快地发展!
